# Supplementary material for: Mitigating Effects of Tanacetum balsamita L. on Metabolic Dysfunction-Associated Fatty Liver Disease (MAFLD)
Source: Plants (Basel). 2024 Jul 27;13(15):2086. doi: 10.3390/plants13152086 (PMC11314425; doi:10.3390/plants13152086)
Supplement: Supplementary file 1 [file plants-13-02086-s001.zip › plants-3089883-supplementary.pdf]

Mitigating Effects of *Tanacetum balsamita* L. on Metabolic Dysfunction-Associated Fatty Liver Disease (MAFLD)

Rositsa Mihaylova [1], Reneta Gevrenova [2,*], Alexandra Petrova [1], Yonko Savov [3], Dimitrina Zheleva-Dimitrova [2], Vessela Balabanova [2], Georgi Momekov [1] and Rumyana Simeonova [1]

1 Department of Pharmacology, Pharmacotherapy and Toxicology, Faculty of Pharmacy, Medical University of Sofia, Sofia 1000, Bulgaria; rmihaylova@pharmfac.mu-sofia.bg (R.M.); aleksandrapetrova91@yahoo.com (A.P.); gmomekov@pharmfac.mu-sofia.bg (G.M.); rsimeonova@pharmfac.mu-sofia.bg (R.S.)

2 Department of Pharmacognosy, Faculty of Pharmacy, Medical University of Sofia, Sofia 1000, Bulgaria; dzheleva@pharmfac.mu-sofia.bg (D.Z.-D.); vbalabanova@pharmfac.mu-sofia.bg (V.B.)

3 Institute of Emergency Medicine "N. I. Pirogov", Bul. Totleben 21, Sofia 1000, Bulgaria; yonko_savov@hotmail.com

* Correspondence: rgevrenova@pharmfac.mu-sofia.bg

Table S1. Weekly body weight changes, presented in grams

Body mass changes (grams)								
Groups	week 1	week 2	week 3	week 4	week 5	week 6	week 7	week 8
control	155.1±4.12	155.1±3.82	156.2±1.62	160.3±1.31	164.2±1.52	163.1±1.61	164.2±1.82	170.3±2.11
ETBhd	160.2±1.62	158.9±4.11	158.8±2.12	163.2±1.61	165.9±4.21	172.2±3.12*	175.9±2.12*	180.1±1.21*
MAFLD	161.2±5.31	165.8±3.80*	170.3±2.21*	165.6±2.42*	161.8±3.22	165.6±2.83	162.2±2.21	160.2±1.12*
MLD+Ats	156.3±4.22	153.8±2.32+	160.1±2.23+	161.8±5.81	158.1±2.21	160.2±2.91+	161.9±3.81	164.8±2.01+
MLD+Acarb	162.1±3.91	168.2±1.51	170.2±2.14	172.2±1.41+	167.9±2.11+	167.8±2.32	165.9±1.32+	170.2±2.11+
MLD+ETBld	160.1±2.11	164.1±1.63	164.9±1.71+	163.8±2.21	166.2±1.22+	167.4±1.62	169.1±4.11+	170.4±5.12+
MLD+ETBhd	163.8±2.22	166.2±1.21	167.7±3.11	167.9±1.32	170.2±2.21+	171.2±4.12	172.1±3.31+	174.9±2.22+

*p≤ 0.005 vs control; **p≤ 0.001 vs control; +p≤ 0.005 vs MAFLD; ++p≤ 0.001 vs MAFLD. Results are expressed as mean ± SD (n=5). The significance of the data was assessed using the Bonferroni-corrected Mann–Whitney U test. Values of p≤ 0.005 were considered statistically significant;

Table S2. Weekly changes in serum triglyceride level (mmol/L)

Blood triglycerides level (mmol/L)								
	week 1	week 2	week 3	week 4	week 5	week 6	week 7	week 8
control	0.40±0.08	0.41±0.19	0.42±0.18	0.43±0.28	0.38±0.22	0.42±0.09	0.42±0.26	0.40±0.02
ETBhd	0.48±0.02	0.52±0.01	0.36±0.01	0.33±0.02	0.31±0.01	0.51±0.01	0.32±0.02	0.33±0.02**
MAFLD	0.42±0.04	0.64±0.05*	0.86±0.06*	1.12±0.08*	1.21±0.09**	1.81±0.12**	2.41±0.08**	2.81±0.16**
MLD+Ats	0.44±0.08	0.48±0.19	0.52±0.19	0.62±0.22++	0.72±0.18++	0.79±0.13++	0.84±0.09++	0.92±0.09++
MLD+Acarb	0.48±0.09	0.52±0.09+	0.54±0.08+	0.61±0.11++	0.77±0.14++	0.84±0.12++	1.21±0.14++	1.82±0.16++

MLD+ETBld	0.42±0.03	0.48±0.02^{+}	0.51±0.01^{+}	0.70±0.02^{++}	0.78±0.02^{++}	0.91±0.02^{++}	1.21±0.03^{++}	1.61±0.15^{++}
MLD+ETBhd	0.51±0.02^{+}	0.52±0.03^{+}	0.62±0.12^{+}	0.72±0.18	0.81±0.19^{++}	0.86±0.28^{++}	1.22±0.29^{++}	1.22±0.12^{++}

*p≤ 0.005 vs control; **p≤ 0.001 vs control; $^{+}$p≤ 0.005 vs MAFLD; $^{++}$p≤ 0.001 vs MAFLD . Results are expressed as mean ± SD (n=5). The significance of the data was assessed using the Bonferroni-corrected Mann – Whitney U test. Values of p≤ 0.005 were considered statistically significant;

Table S3. Weekly changes in blood cholesterol level (mmol/L)

Blood cholesterol level (mmol/L)								
	week 1	week 2	week 3	week 4	week 5	week 6	week 7	week 8
control	1.2±0.11	1.3±0.14	1.6±0.15	1.5±0.12	1.2±0.14	1.4±0.21	1.3±0.18	1.4±0.12
ETBhd	1.5±0.12**	1.5±0.08	1.6±0.12	1.3±0.14	1.4±0.11	1.6±0.06	1.5±0.12	1.6±0.11
MAFLD	1.2±0.15	1.6±0.12	2.4±0.08**	2.8±0.14**	3.1±0.12**	3.6±0.22**	3.8±0.14**	4.1±0.12**
MLD+Ats	1.3±0.21	1.8±0.15	1.9±0.17^{++}	2.4±0.12^{++}	2.4±0.10^{++}	2.2±0.12^{++}	2.1±0.16^{++}	2.2±0.08^{++}
MLD+Acarb	1.2±0.12	1.2±0.08^{++}	2.1±0.11^{++}	2.0±0.15^{++}	2.2±0.12^{++}	2.4±0.14^{++}	2.8±0.17^{++}	3.2±0.13^{++}
MLD+ETBld	1.5±0.21	1.4±0.12	1.8±0.14^{++}	2.2±0.11^{++}	2.4±0.14^{++}	2.8±0.17^{++}	2.6±0.09^{++}	2.4±0.08^{++}
MLD+ETBhd	1.6±0.22	1.8±0.11	2.2±0.13	2.4±0.17^{+}	2.6±0.08^{++}	2.8±0.09^{++}	2.4±0.12^{++}	2.4±0.14^{++}

*p≤ 0.005 vs control; **p≤ 0.001 vs control; $^{+}$p≤ 0.005 vs MAFLD; $^{++}$p≤ 0.001 vs MAFLD. Results are expressed as mean ± SD (n=5). The significance of the data was assessed using the Bonferroni-corrected Mann – Whitney U test. Values of p≤ 0.005 were considered statistically significant;

Table S4. Weekly changes in blood glucose level (mmol/L)

Blood glucose level (mmol/L)								
	week 1	week 2	week 3	week 4	week 5	week 6	week 7	week 8
control	6.2±0.42	6.1±0.51	5.8±0.31	5.6±0.19	6.4±0.45	6.2±0.31	6.6±0.31	6.5±0.41
ETBhd	5.8±0.41	5.6±0.31	6.2±0.21	6.6±0.29**	6.7±0.22	6.9±0.22*	6.8±0.32	7.1±0.36
MAFLD	6.2±0.32	7.1±0.22*	8.2±0.42**	9.2±0.32**	9.2±0.33**	9.6±0.21**	9.8±0.26**	10.2±0.21**
MLD+Ats	5.8±0.42	7.2±0.42	8.2±0.21	8.9±0.22	9.1±0.13	9.1±0.38	9.3±0.28^{+}	9.6±0.19^{+}
MLD+Acarb	5.6±0.33	7.6±0.31	7.8±0.31	9.2±0.38	8.6±0.22	8.3±0.56^{+}	8.1±0.31^{++}	7.8±0.38^{++}
MLD+ETBld	5.8±0.13	7.4±0.21^{++}	8.2±0.22	8.6±0.41	8.8±0.19	8.4±0.39^{++}	8.2±0.39^{++}	8.6±0.31^{++}
MLD+ETBhd	6.2±0.32	7.2±0.22^{+}	7.8±0.42	8.2±0.22^{++}	8.2±0.11^{++}	8.1±0.47^{++}	8.3±0.23^{++}	8.4±0.32^{++}

*p≤ 0.005 vs control; **p≤ 0.001 vs control; $^{+}$p≤ 0.005 vs MAFLD; $^{++}$p≤ 0.001 vs MAFLD. Results are expressed as mean ± SD (n=5). The significance of the data was assessed using the Bonferroni-corrected Mann – Whitney U test. Values of p≤ 0.005 were considered statistically significant;

Abbreviations: ETBld, extract of T. balsamita, low dose (150 mg/kg); ETBhd, extract of T. balsamita high dose (300 mg/kg); MAFLD, metabolic dysfunction-associated fatty liver disease; MLD+Ats, metabolic dysfunction-associated fatty liver disease+atorvastatin; MLD+Acarb, metabolic dysfunction-associated fatty liver disease+acarbose; MLD+ETBld, metabolic dysfunction-associated fatty liver disease + extract of T. balsamita, low dose (150 mg/kg); MLD+ETBhd, metabolic dysfunction-associated fatty liver disease + extract of T. balsamita, high dose (300 mg/kg);
